# Selection of mungbean (*Vigna radiata* L.) mutants with respect to seasonal variation of summer and spring using discriminant function analysis

**DOI:** 10.1016/j.heliyon.2024.e31331

**Published:** 2024-05-15

**Authors:** Muhammad Ali, Muhammad Mahran Aslam, Muhammad Abu Bakar Jaffar

**Affiliations:** Nuclear Institute of Agriculture (NIA), Tandojam, 70060, Sindh, Pakistan

**Keywords:** Mungbean, Discriminant function analysis, Selection, Mutants, Seasons, Breeding

## Abstract

The current study was carried out at Nuclear Institute of Agriculture, Tandojam to assess ten mungbean mutants together with one check cultivar in two separate cropping seasons. The findings revealed that all mutants, with the exception of the branches per plant, had significantly different examined traits. By characterizing ten variables, including plant height (cm), number of branches/plant, number of seeds per pod, grain yield per plot (g/plot), grain weight per plant, pod length (cm), pods per plant, days to flowering, above ground biological weight per plot (g/plot) and days to maturity, the results could considerably differentiate between low and high producing mutants. Discriminant analysis was used to choose high-yielding genotypes. The discriminant score demonstrated a significant canonical correlation of 0.994** and could account for 98.8 % of differences in mungbean production. According to the results of discriminant function analysis, the most significant features are pod length, days to flowering, plant height and above ground biological weight. The highest discriminant scores were displayed by the genotypes AEM66, AEM27, AEM25 and AEM14, identified as high yielding mutants. The low yielding mungbean mutants, designated Viz, AEM20, AEM30, AEM35, AEM-96, AEM29, AEM40 and AEM32 are those that exhibit the lowest values of the discriminant score. Mungbean cultivation is more successful in the summer than it is in the spring.

## 1 Introduction

The mungbean, also known as green gram, was originated on the Indian subcontinent [1]. Mungbean has evolved from a minor, semi-domesticated crop to one of the most significant grain legumes in Asia starting around 1970. Nowadays, about 6 million hectares are planted with mungbean, mostly in South and Southeast Asia but also rising in Australia, the US, Canada [2]. It provides inexpensive sources of protein and carbs that are simple to digest, as well as folate and iron, two nutrients that are frequently in limited supply in developing nations [3]. Mungbean is a crucial part of crop rotations due to its ability to fix nitrogen in the soil as well as its short crop life and low water needs [4]. Cereals sown after mungbeans have the potential to yield more [5]. Pulses are not produced in large enough quantities in Pakistan; imports are used to make up the difference. The deficiency of creative crop development initiatives and seed distribution networks is one of the causes of Pakistan's low pulses production and yield [6]. During 2021-22, 301 thousand hectares of mungbean are grown in the spring and kharif seasons with production of 218 thousand tons accounts for 16 % of country's total pulses production [7]. Significant efforts have been undertaken in the last ten years to create superior cultivars of mungbean. Nevertheless, in order to make mungbean a viable crop and meet the nation's needs for a balanced, nutrient-rich diet, high-yielding cultivars must be chosen and developed [8].

Mungbean landraces yield just 0.4 t/ha or less, whereas developed varieties can yield more than 2 tons/ha [9]. Globally, the main objective of the mungbean breeding program is to breed for high production potential, which can only be accomplished when the material available to breeder demonstrates a significant level of genetic variation [10]. Plant mutation breeding is a technique for creating novel crop types by chemical or physical radiation [11]. Identification of plant mutants with desirable traits requires a valid screening mechanism to increase agricultural productivity [2]. Genetic diversity study and correlation data may help in the selection of genotypes with desirable values of variables of breeding importance, such as maturity, pod features, and grain size [12]. Breeders suggest utilizing selection indices as an alternative method for selecting the desired high yielding genotypes. Numerous studies have used selection indices to identify the most valuable genotypes and the best combinations of traits with the goal of subtly increasing yield in various plants [13,14]. The most well-known selection indices use discriminant functions based on the relative economic relevance of certain traits [15]. The discriminant analysis offers an equation that allows for the greatest possible separation of high- and low-yield genotypes [16]. In addition to examining multivariate differences between groups, it can also be used to identify which variables are most effective at differentiating between groups [15].

The objective of the current study was to construct a multivariate statistical model that would support the holistic selection criterion of mutant variation in addition to yield data for upcoming mungbean breeding initiative and seasonal effect analyses on the crop's growth.

## 2 Material and methods

### 2.1 Experimental field conditions

**Fig. 1:**
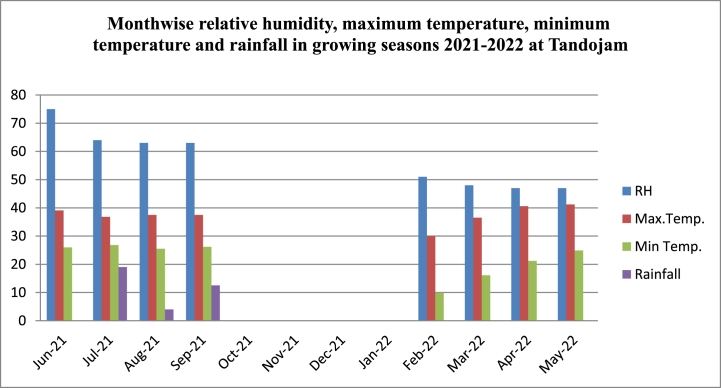
Monthwise relative humidity, maximum temperature, minimum temperature and rainfall in growing seasons 2021–2022 at Tandojam.

The experiments were carried out at the experimental farm of Nuclear Institute of Agriculture (Latitude: 25°N and Longitude 68°E) Tandojam, Pakistan. The weather data of summer 2021 and spring 2022 is collected from Regional agro meteorological department, Tandojam where average rainfall were about 7.66 mm in summer 21, with maximum temperature of 37.8 °C, minimum temperature of 26.1 °C and relative humidity of 67.33 %. Meanwhile, spring 2022 showed average maximum temperature of 35.7 °C, minimum temperature of 15.73 °C, 48.66 % relative humidity and 0 mm rainfall. Monthwise data of both seasons on the relative humidity, maximum temperature, minimum temperature and rainfall is shown in Fig. 1.

### 2.2 Plant materials

The plant material consisted of ten advanced mutants (M_6_) of mungbean, which were tested along with one check commercial cultivar AEM-96. (All plant material (*Vigna radiata*-mungbean mutants) experimental research and field studies had met the terms of institutional, national and international guidelines and legislation).

### 2.3 Experimental design and crop management

Genotypes were grown in a randomized complete block design with three replications. The plot area was 2.7 m^2^, including 3 rows of 3 m long, spaced at 30 cm. Dibbler sowing was done in summer-2021 and spring-2022 in the months of June and February respectively. Thinning was done to maintain plant to plant distance for optimum growth of crop. Recommended agronomic practices were carried out as per scheduled of [17].

## 3 Data collection

Crop was harvested at ninety percent physiological maturity of pods during early hours in the mornings. Three plants from each plot per replication were selected randomly to determine the following traits: plant height (cm), branches per plant, pod length (cm), pods per plant, seed per pod, grain weight (g/plant), yield per plot (g/plot), days to flowering (when 50 % of the plants in a plot were at the flowering stage), days to maturity (when 90 % plants in a plot attained maturity) and above ground biological weight per plot (g/plot).

### 3.1 Statistical analysis

Analysis of variance (ANOVA) of the investigated characters in randomized complete block design was conducted using Statistix 8.1. Means were compared with check AEM-96 using Dunnets test at probability level of 5 %. The combined analysis of variance (summer-21 and spring-22) and Pearson's correlation coefficients were computed using Statistix 8.1.

Linear discriminant function analysis of ten mungbean mutants and one check cultivar (Table 1) with the ten traits was conducted using IBM SPSS statistics version 26 provided all genotypes were divided into two groups based on average seed yield over 2 seasons. Group one had included four high yielding genotypes and group two contained remaining seven low yielding genotypes (Table 7; Fig. 3). Discriminant function analysis provides a formula for maximal separation or discriminating between two genotype groups. Additionally, the following were the key concepts used to describe DFA in our study:

**Table 1:** Genotype code, name, types and source.

Sr. no.	Genotype code	Genotype name	Station	Type	Source
1	V1	AEM32	NIA, Tandojam	Mutant	Co-60 (200 Gy)
2	V2	AEM14	NIA, Tandojam	Mutant	Co-60 (200 Gy)
3	V3	AEM27	NIA, Tandojam	Mutant	Co-60 (300 Gy)
4	V4	AEM29	NIA, Tandojam	Mutant	Co-60 (300 Gy)
5	V5	AEM40	NIA, Tandojam	Mutant	Co-60 (300 Gy)
6	V6	AEM25	NIA, Tandojam	Mutant	Co-60 (200 Gy)
7	V7	AEM30	NIA, Tandojam	Mutant	Co-60 (300 Gy)
8	V8	AEM35	NIA, Tandojam	Mutant	Co-60 (300 Gy)
9	V9	AEM20	NIA, Tandojam	Mutant	Co-60 (200 Gy)
10	V10	AEM66	NIA, Tandojam	Mutant	Co-60 (200 Gy)
11	V11	AEM-96	NIA, Tandojam	Check	Check

NIA=Nuclear Institute of Agriculture, Tandojam, Sindh, Pakistan.

Predictors (independent variables) are used to make judgments. Dependent variable consisted of two groups of ten mutants and one check cultivar. Discriminant function is produced by combining discriminating (independent) variables in a linear fashion and conceptualized as a multiple regression equation [18] as given below:

(1) $$D^{2}=a+b_{1}x_{1}+b_{2}x_{2}+\ldots \ldots \ldots \ldots \ldots \ldots \ldots \ldots ..+b_{n}x_{n}$$

D^2^ = Discriminant score or Discriminant function, “a” an intercept, “b” discriminant coefficients and “x” discriminating variables.

A standardized discriminant coefficient (b_1_ to b_n_) for each variable in each discriminant function show how much each variable contributes to group discrimination. The bigger the coefficient, the more important the given variable was. The canonical correlation (the relationship between the discriminant function and ten independent variables) offers a measure of model fit, interpreted as the percentage of variation explained. Wilk's lambda statistic, examines the relevance of discriminant function, has a value between 0 and 1. When it is close to zero and significant, it indicates that the DFA has a decent ability to distinguish between the genotypes of two groups and vice versa. As a result, it provides information on the variance of the dependent variable (two groups of 10 mutants and one check cultivar) that is not provided by the discriminant function.

The normalized canonical discriminant function coefficients show comparative weights of every variable to the corresponding discriminating function. Structure matrix is another method of determining the interaction between groups of genotypes and discriminant functions. In the end, we obtain discriminant scores, which are the total of a weighted linear combination of the discriminating factors as follows:

(2) $$D^{2}=14.8\;\text{GW}+11.1\;Y/P+8.9\;B/P+4.1\;\text{PH}+2.6\;\text{FD}+0.98\;S/P\;‐\;6.4\;P/P\;‐\;9.7\;\text{PL}\;‐18.0\;\text{BW}$$

(where D^2^ is the discriminant score, PH plant height, B/P branches per plant, P/P pods per plant, PL pod length, S/P seed per pod, Y/P yield per plot, GW grain weight per plant, FD days to flowering, MD days to maturity and BW above ground biological weight per plot).

In our analysis, we ranked genotypes based on the discriminant scores (Eq-2).

The widespread application of discriminant analysis is subject to several restrictions (e.g., the requirement to identify groups prior to analysis and the difficulty of meeting its strong assumptions).

## 4 Results

The findings demonstrated that the coefficient of variation (CV %) for each trial was less than 20 %, allowing for the application of a combined analysis [19].

### 4.1 Mean performance

**Fig. 2:**
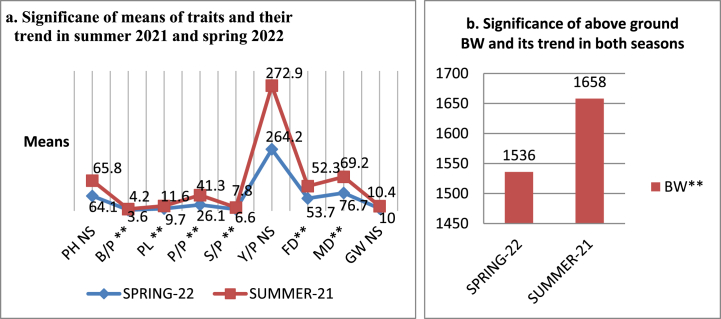
Effect of seasons on the morphological traits (a & b) of mungbean mutants Abbreviations: PH, plant height (cm); B/P, branches per plant; P/P, pods per plant; PL, pod length(cm); S/P, seed per pod; Y/P, yield per plot (g/plot); GW, grain weight per plant(g/plant); FD, days to flowering; MD, days to maturity; BW, above groung biological weight per plot; NS, non-significant; ** highly significant.

With the exception of plant height, grain weight, and yield per plot, the mean values for every trait under analysis demonstrated the highly significant influence of both seasons. As seen in Fig. 2, it was found that the mean values were greater in the summer than in the spring.

**Fig. 3:**
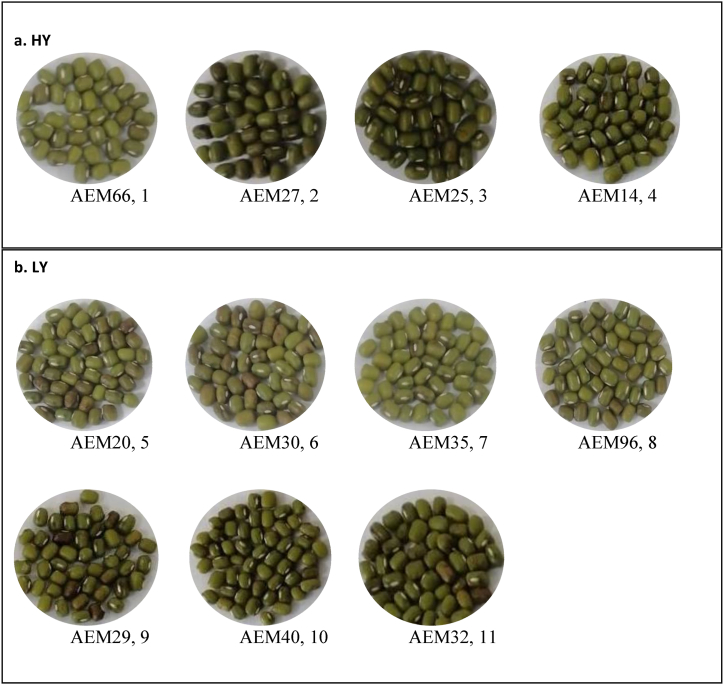
Phenotypic variability in the seed of the mungbean mutants; a. High yielder (HY) and b. Low yielder (LY). Each picture is labeled with mutant name and its ranking position; 1 to 11 by discriminator score. The seeds were kept in a single plane for taking photographs to highlight the seed size variability.

Table 2, Table 3, Table 4 provide the average yield and character data for the mutants along with check AEM-96. The findings showed that there were significant differences in all studied characters for genotypes (mutants) except for branches per plant. The plant height varied from 58.7 cm for AEM20 to 73.8 cm for AEM66 (as an average of the two seasons). AEM-96, had the 4.05 branches per plant in both seasons, with no variety had outperformed the check cultivar statistically. For the pods per plant attribute, there were highly significant variations among genotypes, seasons, and their interactions (SxG). In both seasons, the average number of pods per plant for three mutants AEM14, AEM27 and AEM25 were statistically higher than check cultivar. Mutant AEM14 produced the highest number of pods with 51.99, followed by AEM27 with 50.88 pods per plant in the summer of 2021. While in spring-22, mutant AEM30 with 29 pods per plant was at top, followed by AEM35 and AEM20 with 28.21 pods per plant (Table 2).

**Table 2:** Mean performances of mungbean mutants for morphological characters across the growing seasons 2021–2022.

Characters	PH			B/P			P/P		
Genotypes	SUM-21	SPR-22	Mean	SUM-21	SPR-22	Mean	SUM-21	SPR-22	Mean
AEM32	65.8	63.6	64.7	4.22	3.33	3.78	44.34	19.43	31.88
AEM14	70.7	59.7	67.6	4.11	3.67	3.89	51.99	23.53	37.76
AEM27	72.8	67.7	69.8	4.22	3.89	4.05	50.88	25.07	37.97
AEM29	67.6	61.7	66.1	3.56	3.78	3.67	45.69	25.73	35.71
AEM40	68.3	68.7	68.0	5.44	3.89	4.66	34.47	26.57	30.52
AEM25	61.0	64.3	61.8	4.00	3.44	3.72	44.90	30.22	37.56
AEM30	65.9	63.0	65.6	4.22	3.89	4.05	37.66	29.80	33.73
AEM35	59.3	59.7	62.0	4.33	3.89	4.11	32.67	28.21	30.44
AEM20	54.4	61.3	58.7	4.33	3.44	3.88	38.46	28.21	33.33
AEM66	77.8	70.0	73.8	3.78	3.22	3.5	36.67	24.67	30.67
AEM-96	60.8	66.0	63.7	4.44	3.67	4.05	36.67	25.11	30.89
**Mean**	**65.8**	**64.1**	**65.6**	**4.2**	**3.65**	**3.9**	**41.3**	**26.1**	**33.7**
**Season (S)**	**NS**			b			b		
**Genotype (G)**	a			**NS**			b		
**S x G**	**NS**			**NS**			b		
**CV%**	**10.69**			**16.62**			**10.70**		

Abbreviations: PH, plant height (cm); B/P, branches per plant; P/P, pods per plant; NS, non-significant; SUM-21, summer 2021; SPR-22, spring 2022.

a Significant result at 0.05.

b Highly significant result at 0.01

**Table 3:** Mean performances of mungbean mutants for morphological characters across the growing seasons 2021–2022.

Characters		PL			S/P			Y/P	
Genotypes	SUM-21	SPR-22	Mean	SUM-21	SPR-22	Mean	SUM-21	SPR-22	Mean
AEM32	8.03	6.68	7.36	11.11	9.56	10.33	242.7	227.6	235.1
AEM14	7.66	6.69	7.17	12.64	10.56	11.60	326.0	302.7	314
AEM27	8.08	7.03	7.56	10.73	10.22	10.47	353.6	270.8	312
AEM29	8.06	6.04	7.05	11.45	9.01	10.23	213.2	273.2	243.2
AEM40	7.54	6.09	6.82	11.06	9.56	10.31	221.8	233.1	227.4
AEM25	7.94	6.93	7.44	11.03	9.56	10.29	336.2	336.7	336
AEM30	7.83	6.66	7.24	11.09	9.22	10.16	308.9	209.2	259.0
AEM35	7.78	6.84	7.31	12.15	10.22	11.19	178.5	263.6	221.1
AEM20	7.70	6.41	7.06	11.64	9.22	10.43	254.5	282.0	268.3
AEM66	8.03	7.01	7.52	13.09	9.67	11.38	366.1	265.8	316
AEM-96	7.40	6.14	6.77	11.42	9.89	10.66	200.3	242.1	221.2
**Mean**	**7.8**	**6.6**	**7.2**	**11.6**	**9.7**	**10.6**	**272.9**	**264.2**	**268.6**
**Season (S)**	b			b			**NS**		
**Genotype (G)**	a			b			b		
**S x G**	**NS**			**NS**			b		
**CV%**		**5.87**			**6.61**			**17.10**	

Abbreviations: PL, pod length (cm); S/P, seed per pod; Y/P, yield per plot (g/plot); NS, non-significant; SUM-21, summer 2021; SPR-22, spring 2022.

a Significant result at 0.05.

b Highly significant result at 0.01

**Table 4:** Mean performances of mungbean mutants for morphological characters across the growing seasons 2021–2022.

Characters	GW			FD			MD			BW		
Genotypes	SUM-21	SPR-22	Mean	SUM-21	SPR-22	Mean	SUM-21	SPR-22	Mean	SUM-21	SPR-22	Mean
AEM32	13.04	12.54	12.7	54.00	54.00	54.00	70.00	78.00	74.00	1473	1350	1411
AEM14	9.46	9.17	9.31	50.00	54.00	52.00	67.00	76.00	71.50	1837	1633	1735
AEM27	8.43	8.30	8.36	51.00	55.00	53.00	69.00	77.00	73.00	1707	1533	1620
AEM29	7.63	7.53	7.58	51.33	53.67	52.50	67.33	76.00	71.67	1538	1516	1527
AEM40	17.95	17.48	17.7	55.33	54.67	55.00	73.00	75.00	74.00	1340	1366	1353
AEM25	7.97	8.53	8.25	49.67	51.33	50.5	66.33	75.67	71.0	1707	1700	1703
AEM30	7.28	6.91	7.09	51.33	52.67	52.00	69.00	77.67	73.33	1766	1233	1499
AEM35	14.42	14.31	14.3	51.00	56.00	53.50	71.33	77.67	74.50	1475	1600	1537
AEM20	9.20	8.71	8.95	53.00	53.00	53.00	67.33	76.67	72.00	1436	1466	1451
AEM66	10.27	8.96	9.61	53.00	53.67	53.33	68.67	76.00	72.33	2130	1716	1923
AEM-96	8.40	7.72	8.06	55.33	52.67	54.00	72.67	78.33	75.50	1830	1783	1806
**Mean**	**10.4**	**10.0**	**10.2**	**52.3**	**53.7**	**52.9**	**69.2**	**76.7**	**72.9**	**1658**	**1536**	**1597**
**Season (S)**	**NS**			b			b			b		
**Genotype (G)**	b			b			b			b		
**S x G**	**NS**			b			a			**NS**		
**CV%**	**11.4**			**3.35**			**2.54**			**12.63**		

Abbreviations: GW, grain weight per plant (g); FD, days to flowering; MD, days to maturity; BW, above ground biological weight (g/plot); SUM-21, summer 2021; SPR-22, spring 2022. NS, non-significant.

a Significant result at 0.05.

b Highly significant result at 0.01

Data in Table 3 showed that pod length varied considerably depending on genotypes and seasons. The interactions of the season with genotypes differed non-significantly. The mean pod length for the summer season was higher than the mean for the spring. The check AEM-96 showed the smallest pod length of 6.77 cm and three mutants AEM27, AEM66 and AEM25 showed significantly larger pod length against a mean value of 7.2 cm over the two seasons.

There are highly significant differences between the genotypes and seasons for the seed per pod (Table 3). It varied from the lowest mean value of 9.5 and 10.73 to highest of 10.56 and 13.09 seed per pod in the spring and the summer respectively. The check cultivar, the AEM-96, got the mean value (10.66) in both seasons with non-significant differences from all other mutants.

The results regarded yield per plot depicted that in two seasons, the mutants viz. AEM25, AEM66, AEM14 and AEM27 significantly produced more yield per plot with 336, 316, 314, and 312 (g/plot) respectively against 221.2 of check AEM-96. In summer 21, the AEM66 and AEM27 produced the highest yields of 366.1 and 353.6 g/plot, while in spring 22, the mutants AEM25 and AEM14 produced the highest of 336.7 and 302.7.

The results for grain weight (Table 4) revealed highly significant differences among mutants, while the seasons and the SxG interaction showed no statistical difference. The grain weight of AEM40 was higher than other mutants and check cultivar for both the seasons (17.95g & 17.48g). In contrast to other mutants, AEM30 showed the lowest values of grain weight (7.28g & 6.91g) for both the seasons. According to the mean values of grain weight, the summer season favored greater grain production than the spring season (Table 4). Additionally, the mutants AEM35 and AEM32 showed greater seed output than check AEM-96.

The results showed that there were highly significant variations in the days to flowering for different seasons, genotypes, and their interactions. The only mutant AEM25 significantly flowered earlier (50.5days) than check AEM-96 (54.0days) in both seasons.

Days to maturity demonstrated extremely significant differences between seasons, genotypes, and significant differences to their interactions. Five mutants viz. AEM14, AEM29, AEM25 AEM20 and AEM66 matured earlier than check AEM-96.

Highly significant differences between genotypes and seasons for the above ground biological weight per plot was observed (Table 4) however their interactions (SxG) were non-significant. With notable differences from the check cultivar AEM-96, the AEM32, AEM40, AEM30 and AEM20 mutants got the lower mean values of BW over the course of both seasons.

Table 5 shows the statistically significant means compared to the check AEM-96 using Dunnett's test. Three varieties (AEM14, AEM27, and AEM25) had considerably more pods per plant than the other varieties. Pod length significantly increased in three mutants (AEM27, AEM25 & AEM66). Four genotypes (AEM14, AEM27, AEM25, and AEM66) demonstrated noticeably higher output per plot compared to the check. More grains per plant were generated by three genotypes (AEM32, AEM40, and AEM35). One of the varieties (AEM25) bloomed and reached maturity a lot sooner than AEM-96. Four varieties (AEM32, AEM40, AEM30 and AEM20) substantially decreased the amount of above-ground biological weight per plot compared to the check. In general, understanding of genetic variability and the nature of characters association aids in the creation of effective multiple trait selection schemes [20]. Phenotypic variability in the seed of the mungbean mutants is shown in Fig. 3.

**Table 5:** Dunnett's statistically significant means of traits against check AEM-96.

Genotype	P/P	PL	Y/P	GW	FD	MD	BW
**V1** (AEM32)	31.8	7.3	235.1	**12.7**^a^	54	74	**1411.7**^a^
**V2** (AEM14)	**37.7**^a^	7.1	**314.3**^a^	9.3	52	**71.5**^a^	1735.5
**V3** (AEM27)	**37.9**^a^	**7.5**^a^	**312.2**^a^	8.3	53	73	1620.6
**V4** (AEM29)	35.7	7.0	243.2	7.5	52.5	**71.6**^a^	1527.4
**V5** (AEM40)	30.5	6.8	227.4	**17.7**^a^	55	74	**1353.8**^a^
**V6** (AEM25)	**37.5**^a^	**7.4**^a^	**336.4**^a^	8.2	**50.5**^a^	**71**^a^	1703.6
**V7** (AEM30)	33.7	7.2	259.0	7.1	52	73.3	**1499.7**^a^
**V8** (AEM35)	30.4	7.3	221.1	**14.3**^a^	53.5	74.5	1537.5
**V9** (AEM20)	33.3	7.0	268.3	8.9	53	**72**^a^	**1451.4**^a^
**V10** (AEM66)	30.6	**7.5**^a^	**316.0**^a^	9.6	53.3	**72.3**^a^	1923.8
**V11 (AEM-96)**	30.8	6.7	221.2	8.1	54	75.5	1806.7
**D**_**DUNNETT**_	**5.3**	**0.62**	**67.29**	**1.70**	**2.59**	**2.71**	**295.32**

Abbrevaition; P/P, pods per plant; PL, pod length; Y/P, yield per plot (g/plot); GW, grain weight per plant (g/plant); FD, days to flowering; MD, days to maturity and BW, above ground biological weight (g/plot).

a *p* = 0.05.

### 4.2 Pearson's correlation coefficient

The coefficients of correlation were computed between yield and all other traits as illustrated in Table 6. Data showed that yield per plot (Y/P) in summer 2021 was positively associated with the plant height ( 0.496**), above ground biological weight ( 0.490**) and pod length ( 0.420*) while negatively associated with days to maturity (−0.521**) and days to flowering (−0.402*). In spring 2022 yield per plot (Y/P) was associated with the above ground biological weight ( 0.627**). Pooled data of both seasons revealed that yield per plot was highly significantly and positively correlated with pod length (0.530**) and above ground biological weight (0.518**), while it was negatively highly significantly correlated with days to flowering (−0.405**) and days to maturity (−0.495**) respectively.

**Table 6:** Pearson correlation between yield and agronomic traits with significance level.

	Summer-21	Spring-22	Pooled data of both seasons
Characters	Y/P	Y/P	Y/P
PH	**0.496**^a^	0.028	0.289
B/P	−0.243	−0.076	−0.292
P/P	0.318	−0.061	0.246
PL	**0.420**^b^	0.190	**0.530**^a^
S/P	0.190	0.120	0.275
GW	−0.31	−0.085	−0.292
FD	**−0.402**^b^	0.034	**−0.405**^a^
MD	**−0.521**^a^	0.033	**−0.495**^a^
BW	**0.490**^a^	**0.627**^a^	**0.518**^a^

a Correlation is highly significant at the 0.01 level.

b Correlation is significant at the 0.05 level. Abbreviations; PH, plant height (cm); B/P, branches per plant; P/P, pods per plant; PL, pod length(cm); S/P, seed per pod; Y/P, yield per plot(g/plot); GW, grain weight per plant(g/plant); FD, days to flowering; MD, days to maturity; BW, above ground biological weight.

**Table 7:** Comparison between two supposed groups using the Wilk's lambda test.

Groups	Traits	PH	B/P	P/P	PL	S/P	Y/P	GW	FD	MD	BW
**Group 1 (Low yielder)**	**Mean**	64.1	4.1	35.8	7.1	10.5	239.4	8.4	53.4	73.6	1863.5
	**Std**	3.0	0.4	8.6	0.2	0.4	18.5	1.7	1.0	1.4	692.1
**Group 2 (High yielder)**	**Mean**	68.3	3.9	34.8	7.4	10.9	319.8	8.4	52.2	72.0	2504.2
	**Std**	5.0	0.2	6.1	0.2	0.7	11.2	1.3	1.3	0.2	333.3
**Wilk's Lambda**		0.8	0.9	1.0	**0.6**^a^	0.8	0.1**	1.0	0.7	0.7	0.8

Abbreviuation; PH, plant height (cm); B/P, branches per plant; P/P, pods per plant; PL, pod length; S/P, seeds per pod; Y/P, yield per plot (g/plot); GW, grain weight per plant (g/plant); FD, days to flowering; MD, days to maturity and BW, above ground biological weight per plot (g/plot).Foot notes bp=0.01

a ***p*****= 0.05**

### 4.3 Discriminant function analysis

The mungbean genotypes were ordered in descending order based on the average yield over two seasons. Seven genotypes were chosen for group one (low yielder genotypes), whereas four genotypes were chosen for group two (high yielder genotypes).

### 4.4 Group equality test

Means, standard deviations and Wilk's lambda test of the investigated characters for two groups are illustrated in Table 7. Using the Wilk's lambda statistic, the results revealed that there were significant variations between the two groups for pod length (0.6*) and yield per plot (0.1**) and moving on with analysis would be meaningful. Meanwhile examining the group means and standard deviations might help in gaining a general understanding of the variables that may be important. Group 1 has small values of mean for all traits except for B/P, P/P, FD and MD as compared to Group 2. Large standard deviations of PH, P/P, Y/P, and BW imply that the data points are far from the mean, but modest standard deviations of B/P, PL, GW, FD, and MD indicate that the data points are closely clustered around the mean in the two hypothetical groups (Groups 1 & 2).

### 4.5 Structure matrix and standardized canonical discriminant function coefficient

DFA not only quantifies the overall distance between mutants using the discriminant score (D^2^), but also identifies the traits that are used to differentiate cultivars within the studied specification. The standardized Canonical coefficients (Table 8) are used to create discriminant function (equation (2). The Significance of discriminant function model is represented by a highly significant small value of Wilks'slambda (0.012**). The structure matrix is regarded as the dividing line between significant and minor variables. The dependent variable separation is primarily influenced by the independent variables with large structure matrix values. In Table 8, a ranking from 1 to 9 indicates the highest to lowest structural matrix values of the researched traits as Y/P, PL, FD, PH, BW, S/P, B/P, P/P and GW respectively. The canonical correlation gauges the strength of the relationship between independent factors and the predicted values (score) fitted by the discriminant function (eleven measured traits). A Canonical correlation of 0.99, as shown in Table 8, indicates that the discriminate function model accounts for 98.1 % of the variance among the ten mutants and one check cultivar.

**Table 8:** Structure matrix and Standardized Canonical discriminant function coefficients.

Characters	PH	B/P	P/P	PL	S/P	Y/P	GW	FD	BW
**Structure matrix**	0.06	−0.03	−0.007	0.090	0.05	0.28	−0.001	−0.06	0.06
**Standardized canonical discriminant function coefficients**	4.10	8.94	−6.44	−9.79	0.98	11.13	14.83	2.6	−18.0
**Rank**	**4**	**7**	**8**	**2**	**6**	**1**	**9**	**3**	**5**
**Canonical correlation**	0.994** (p=0.01)								
**Wilk's Lambda**	0.012** (p=0.01)								

Abbreviations; PH, plant height (cm); B/P, branches per plant; P/P, pods per plant; PL, pod length(cm); S/P, seed per pod; Y/P, yield per plot(g/plot); GW, grain weight per plant(g/plant); FD, days to flowering and BW, above ground biological weight per plot.

**4.6 Classification of mutants and discriminant scores**

Table 9 appeared that genotype AEM40 and AEM35 is a low yield genotype (group 1) and AEM27 as a high yield genotype (group 2) but based on discriminant function, Eq-2 classified AEM40 and AEM35 as group 2 (high yield) and AEM27 as group 1 (low yield). Other genotypes like AEM32, AEM14, AEM29, AEM25, AEM30, AEM20, AEM66 and AEM-96 were classified in their expected groupings. Thus, the correct classification rate was 8/11 = 72.72 %. Phenotypic variability in the seed of the mungbean mutant groups (high yielder and low yielder) is shown in Fig. 3.

**Table 9:** Classification of high and low yield mutants.

Groups	Genotype code	Genotype	Discriminant scores	Rank
**High yielder**	V10	AEM66	12.569	**1**
	V3	AEM27	11.000	**2**
	V6	AEM25	10.964	**3**
	V2	AEM14	9.685	**4**
**Low yielder**	V9	AEM20	−5.339	**5**
	V7	AEM30	−5.759	**6**
	V8	AEM35	−5.932	**7**
	V11	AEM-96	−6.144	**8**
	V4	AEM29	−6.256	**9**
	V5	AEM40	−6.668	**10**
	V1	AEM32	−8.120	**11**

## 5 Discussion

The two-year seasonal dataset revealed that summer-2021 had higher rainfall, relative humidity and favorable temperature swings (Regional meteorological department, Tandojam), made it more suitable for mungbean mutants than of spring 2022. The ideal temperature for mungbean growth and development is 28–30 °C, and the range in which the plant continues to produce seed is 33–35 °C [21], was prevailed during summer of 2021. The relative growth rates of mungbean mutants are improved by higher RH [22] that was predominant in the summer of 2021 because of more rainfall. Springtime crops are typically produced on residual moisture [23] and as there was no rainfall in spring 2022, crop was prone to water stress. Owe to these reasons, summer is seen to be a better growing season than spring for the production of mungbeans, the findings are in accordance with [24].

Creating novel varieties that perform consistently well in diverse environments/seasons is the main goal of most plant breeders. This interaction poses a challenging situation to breeders to choose the best varieties. Elite varieties created for one location or season may not perform similarly in other locations or seasons [25]. The study of such interactions must be taken into consideration during the selection process in order to acquire crop varieties that will perform consistently across environments and seasons [26]. In the present study G × E interactions effect are recorded for all the traits in the form of S x G. Four traits, pods P/P, Y/P, FD and MD had shown significant interaction results. Five traits viz; PH, B/P, PL, S/P and GW showed non-significant results, suggesting that selection criterion may be devised upon them as steady genotypes are those that have non-significant GxE effects [25].

An understanding of genetic variability aids in the creation of effective selection schemes [20]. Four mutants (AEM66, AEM14, AEM27, and AEM25) are found suitable for use in future breeding programs since they had much more pods per plant, a greater yield per plot, and longer pods than the check AEM-96.

Mungbean seed yield is a complex character, affected by environment and secondary yield-contributing characters [27]. Breeding programs, aim to create genotypes with high environmental productivity do so through selection based on association studies. Direct selection for yield, only modest progress could be achieved over an extended period of time, due to its complexity. However, indirect selection through yield components has been demonstrated more effective [28]. Based on pooled data from both seasons, the yield per plot was positively correlated with pod length and above ground biological weight but negatively correlated with days to flowering and days to maturity. So, pod length and above ground biological weight are the important traits, directly linked to the early matured grain yield at harvest. These results are in line with findings of [29].

The discriminant function approach, which develops selection criteria based on a combination of most distinctive traits, reduces the chance of incorrectly assigning genotypes to the appropriate group. It helps the breeder achieve breeding program goals and increase productivity [30]. In present study, two groups of mungbean mutants (high yielder and low yielder) based on the yield was predicted and Wilks’ lambda is used to check the heterogeneity of both groups based on the growth data of morphological traits. Results showed that there were substantial differences between the two groups in terms of pod length and yield per plot, indicating that these might be reliable indicators to distinguish between the two mutant groups and proceeding further with the analysis will be meaningful. The investigation yielded a total accurate classification percentage of 72.72 % for the discriminant function. It exceeded the 50.% proportionate chance threshold.

The proposed selection criterion in this study, could explain 98.1 % of seed yield variation between two groups of mutants. The canonical discriminant coefficients and the structure matrix are the two things to rank the significance of each predictor. With the exception of Y/P, PL, FD, PH, and BW, all of the structural matrix values are clearly modest (less than or equal to 0.056), demonstrating their efficacy as differentiators between groups with high and low seed yields. Wilks’ lambda indicates the significance of the discriminant function. A highly significant function (p = 0.01) provides the proportion of total variability not explained, i.e. it is the converse of the squared canonical correlation. So we have 1.99 % unexplained. Due to their high coefficients (Eq (2)), high Wilks' lambda and favorable significant relationships with yield, the four attributes (PL, FD, PH, and BW) were found to be the most distinctive traits. Selection is therefore focused on these traits can raise the yield of mungbeans. Researchers have previously assessed the importance of these traits to confirm their role in improving yield in green gram [31].

The predicted values of the discriminant function model's fitting are the discriminant score. According to discriminant scores, all genotypes are categorized and ranked into two classes; high and low producing groups. A higher discriminant score represents the high-yielding group.

## 6 Conclusion

Discriminant function analysis, a potent multivariate technique, is used in the cases where groups are predetermined and the assumptions of the analysis easily met. In present study, DFA identified a function model, accounts for 98.8 % of the variance in the mungbean mutants, in different cropping seasons. The genotypes AEM66, AEM27, AEM25, and AEM14 demonstrated the greatest discriminant scores and were identified as high yielding mungbean genotypes. A highly significant small value of Wilk's lambda (0.012) highlights the function's strong ability to differentiate itself when using several useful qualities at once. Given their strong relationships with yield and structural matrix values, traits like PL, FD, PH, and BW may be used in next breeding programs to identify promising mungbean mutants. Higher rainfall, relative humidity and favorable temperature swings made summer more suitable for mungbean mutants as compared to spring.

## Data availability statement

Data will be made available on request to corresponding author.

## Funding

This research did not receive any specific funding.

## CRediT authorship contribution statement

**Muhammad Ali:** Writing – review & editing, Writing – original draft, Methodology, Investigation, Formal analysis, Data curation, Conceptualization. **Muhammad Mahran Aslam:** Writing – review & editing. **Muhammad Abu Bakar Jaffar:** Writing – review & editing, Visualization, Software, Formal analysis.

## Declaration of competing interest

The authors declare the following financial interests/personal relationships which may be considered as potential competing interests:Muhammad Ali reports administrative support and equipment, drugs, or supplies were provided by Nuclear Institute of Agriculture, Tandojam, SIndh, Pakistan.
